# Comparative efficacy of Chinese herbal medicines for dialysis patients with uremic pruritus: A systematic review and network meta-analysis

**DOI:** 10.3389/fphar.2023.1064926

**Published:** 2023-01-17

**Authors:** Ping-Hsun Lu, Chien-Cheng Lai, Ling-Ya Chiu, Jen-Yu Wang, Po-Hsuan Lu

**Affiliations:** ^1^ Department of Chinese Medicine, Taipei Tzu Chi Hospital, Buddhist Tzu Chi Medical Foundation, New Taipei City, Taiwan; ^2^ School of Post-Baccalaureate Chinese Medicine, Tzu Chi University, Hualien, Taiwan; ^3^ Department of Medical Education, MacKay Memorial Hospital, Taipei, Taiwan; ^4^ Department of Medicine, MacKay Medical College, New Taipei City, Taiwan; ^5^ Department of Medical Research, MacKay Memorial Hospital, Taipei, Taiwan; ^6^ Department of Dermatology, MacKay Memorial Hospital, Taipei, Taiwan

**Keywords:** Chinese heral medicine, network meta-analysis, touxie-jiedu decoction, uremic pruritus, xiao-yang_ke-li

## Abstract

**Introduction:** Uremic pruritus is common in dialysis patients and reduces their quality of life. Chinese herbal medicine has been effective in patients with this condition.

**Methods:** We conducted a random-effects network meta-analysis to compare the efficacies of different Chinese herbal medicine treatments for uremic pruritus. Outcome measures including the overall effective rates, visual analog scale scores, C-reactive protein levels, and adverse drug reactions were analyzed.

**Results:** The network meta-analysis retrieved 25 randomized controlled trials. Compared with conventional treatments alone, combination treatments with Xiao-Yang-Ke-Li was the most effective intervention in decreasing visual analog scale scores (mean difference −2.98, 95% mean difference −5.05 to −0.91) and levels of C-reactive protein (mean difference −5.01, 95% mean difference −7.27 to −2.75). Conventional treatment combined with Si-Wu Tang was superior to other therapeutic combinations when overall effective rates were determined. The best visual analog scale scores and overall effective rates were achieved by adjunctive treatment with the Touxie-Jiedu-Zhiyang decoction followed by uremic clearance granules; these treatments were the most beneficial for uremic pruritis.

**Conclusion:** Our network meta-analysis provided the relative efficacies of different adjunctive Chinese herbal formulas. Adjunctive treatment with the Touxie-Jiedu-Zhiyang decoction was the best treatment for improving overall effective rates and reducing visual analog scores of uremic pruritus in dialysis patients.

**Systematic Review Registration:** https://www.crd.york.ac.uk/prospero/display_record.php?RecordID=357656; Identifier: CRD42022357656.

## Introduction

Uremic pruritus (UP) is a common and troublesome complication seen in dialysis patients; it affects the quality of life in about 40% of these patients (Mettang and Kremer, 2015). A previous cohort study revealed an association between UP and poor outcomes in dialysis patients, including overall mortality and infection-related death (Ting et al., 2020). Hence, adequate control of UP is necessary for dialysis patients (Ko et al., 2022). The pathophysiology of UP is complicated; it is associated with various inflammatory mediators, neurotransmitters, and opioids. Treatments for UP, including difelikefalin, gabapentin, antihistamines, and phototherapy, have anti-pruritic effects, although adverse drug reactions (ADR), such as diarrhea, vomiting, somnolence, and dizziness, have been reported for some (Hercz et al., 2020; Trachtenberg et al., 2020; Fugal and Serpa, 2022; Ko et al., 2022). The use of UVB radiation in phototherapy is associated with an increased risk of malignancy (Mettang and Kremer, 2015). Because of the aforementioned ADRs, effective treatments with fewer complications, such as complementary and alternative treatments, are crucial for dialysis patients experiencing UP.

Complementary and alternative treatments for UP have been highlighted in recent studies. Acupuncture was reported to alleviate UP (Hwang and Lio, 2021). Auricular acupressure significantly reduced the visual analog scale (VAS) scores of UP and serum histamine levels in dialysis patients (Yan et al., 2015). Chinese herbal medicine (CHM) also plays an important role in adjunctive treatment for UP. A systematic review reported that uremic clearance granules (UCGs) significantly reduced VAS scores, high-sensitivity C-reactive protein (hsCRP), tumor necrosis factor-α (TNF-α), interleukin (IL)-6, creatinine, blood urea nitrogen (BUN), parathyroid hormone (PTH), intact parathyroid hormone (iPTH), and phosphorus (P) in dialysis patients with UP (Lu et al., 2021). Chinese herbal bath therapies, including Chuanxiong, Baijili, and Dahuang, seemed to effectively relieve UP in dialysis patients (Lu et al., 2020). However, there has been no comparison of the efficacies of different Chinese herbal treatments for UP in dialysis patients. In this study, we conducted a systematic review and network meta-analysis to compare the effects of different CHM interventions and give clinical suggestions based on the different clinical outcomes of patients with UP.

## Methods

### Literature search

Seven major databases, including PubMed, Embase, the Cochrane Library, CINAHL, Chinese National Knowledge Infrastructure, the Airiti Library, and Wanfang were searched from their inception to 1 June 2022 without language restrictions. We used Chinese medicine (including herbal medicine, pill, powder, san, granule, and formula), pruritus, uremia, chronic kidney disease, dialysis, and their synonyms as the MeSH and Emtree search headings with free text words using these terms and their combinations. The search strategy is presented in Supplementary Table S1. We also searched the reference sections of accessed papers manually and contacted known experts in the field to identify other studies. Finally, we inspected unpublished studies from the ClinicalTrials.gov registry (http://clinicaltrials.gov/). This study is registered with PROSPERO (registration number, CRD 42022357656). This systematic review is reported as recommended by the Preferred Reporting Items for Systematic Reviews and Meta-Analyses (PRISMA) extension statement for network meta-analyses.

### Study selection

We included only randomized controlled trials (RCTs). The following inclusion criteria were applied: 1) dialysis patients with UP, 2) oral CHM prescription, and 3) availability of quantitative data about pruritus severity. The following exclusion criteria were applied: 1) Chronic kidney disease (CKD) patients without dialysis; 2) dialysis patients not diagnosed with UP; 3) no oral CHM intervention (such as acupuncture, acupressure, and herbal baths); and 4) control groups receiving additional treatments (such as charcoal tablets or antihistamines). If data were raw or missing, we contacted the investigators by e-mail.

### Data extraction and risk of bias assessment

Preliminarily selected studies were assessed for eligibility for network meta-analysis by two reviewers (C-CL and Pi-HL) based on the aforementioned inclusion criteria. The decisions of the two reviewers were individually recorded and compared, and any disagreement was resolved by a third reviewer (Po-HL). The following information was listed for each selected study: first author, publication year, sample size, patient ages, period of intervention, dosage and frequency of interventions, severity of pruritus, and overall effective rate.

Two reviewers independently assessed the risk of bias of each study with the Cochrane Collaboration’s Risk of Bias 2 tool (Sterne et al., 2019). Five domains were assessed by three levels (low, unclear and high risk of bias) for evaluation of the methodological quality of the selected RCTs. Five domains included randomizing process, deviations from intended intervention, missing outcome data, outcome measurement and selection bias. The overall bias of each study was scored according to qualities of five domains. Discrepancies between reviewers were resolved by a third reviewer.

### Outcome measurement

One primary outcomes (VAS scores) and three secondary outcome (ER, CRP levels, and ADRs) were extracted and analyzed. ER was defined as the number of patients cured of pruritus or with improved symptoms out of the total patients. VAS scores ranged from 1 to 10; higher scores were associated with more severe itching sensations.

### Statistical analysis and software

We measured dichotomous outcomes with ER. Continuous outcomes, such as VAS scores and CRP levels, were estimated as weighted mean differences (WMDs) for comparison between groups. The network meta-analysis, network plot, network forest plot, cluster ranking plot, funnel plot, netleague table, and the surface under the cumulative ranking curve (SUCRA) ranking were carried out or produced using the Network package (White, 2015) of STATA (version MP 17.0, StataCorp, College Station, TX, United States). SUCRA is an index between 0 and 1, and larger SUCRAs are associated with better treatment responses. The risk of bias graph and summary was illustrated by RevMan 5.4 (Cochrane Collaboration, Copenhagen, Denmark).

## Results

### Characteristics of included studies

**FIGURE 1:**
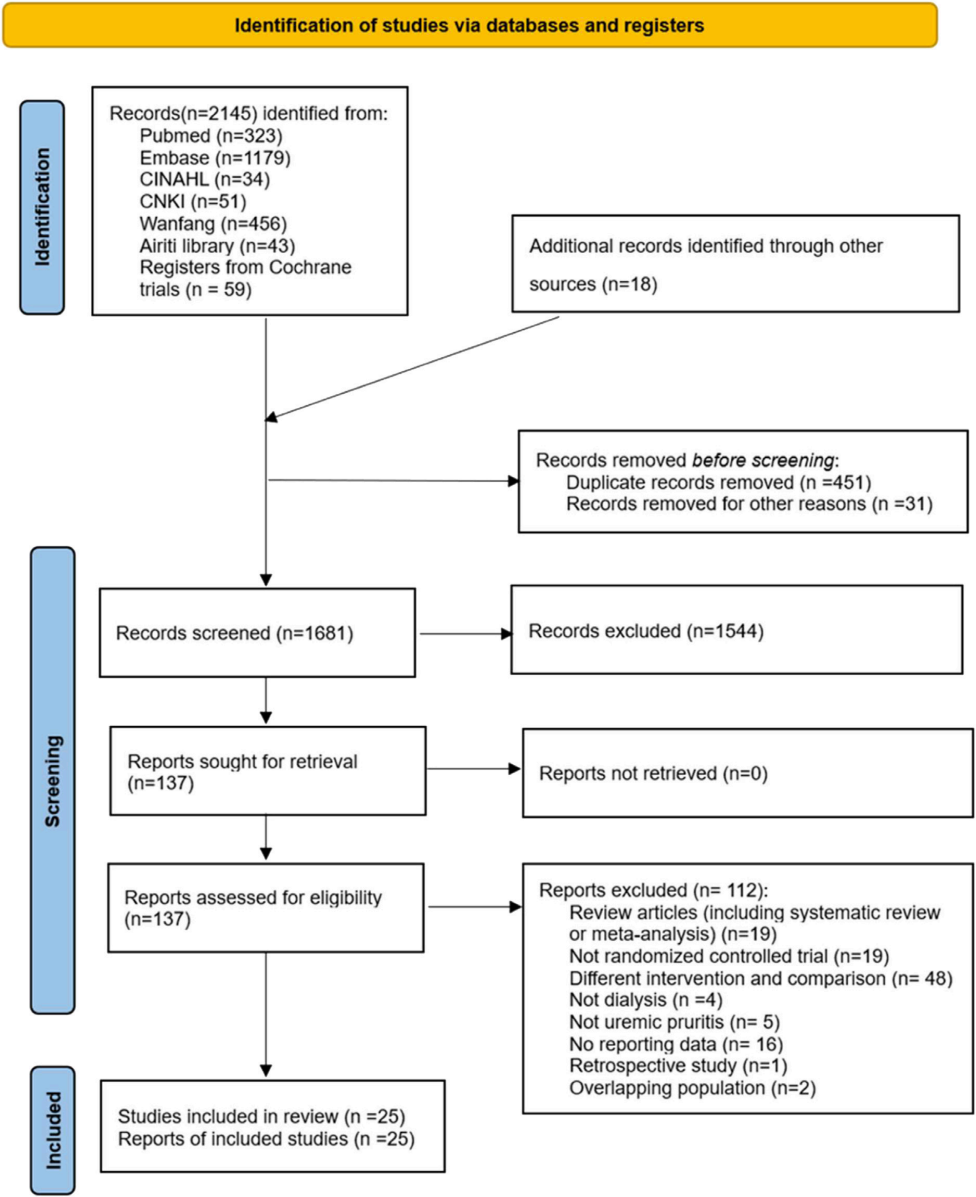
PRISMA 2020 Flow Diagram.

We used a PRISMA flowchart to explain the process of identifying and selecting randomized controlled trials (RCTs) for evaluating the effects of CHM in dialysis patients with UP (Figure 1). We identified 2145 articles from electronic databases and 18 additional records obtained from other sources. We excluded 1544 articles based on their titles and abstracts. We then reviewed the full texts of the remaining 137 articles. We excluded 113 of these articles for the following reasons: 19 studies were review articles, 19 studies were not RCTs, 49 studies involved different interventions (such as acupuncture, acupressure and other Chinese medicine manipulation), five studies did not involve UP patients, eight studies did not report data, two studies involved overlapping populations, and one study was retrospective. We qualitatively and quantitatively synthesized the remaining 25 articles.

The characteristics of the included RCTs are listed in Table 1 and Supplementary Table S3. All trials were published between 2004 and 2021 and included 1703 participants. The sample sizes per study ranged from 32 to 128 participants. All CHM interventions were combined with conventional treatment (dialysis with electrolyte-balanced, bodily fluid and blood pressure maintenance). Of the studies following the CHM treatments of UP patients undergoing dialysis, eight involved UCGs, four assessed patients were treated with the Touxie–Jiedu–Zhiyang decoction (TJZD), and the remaining 13 examined patients were treated respectively with Fan’s TJZD (FanTJZD), Yangxue–Runfu–Yin (YXRFY), Yangxue-Wensheng Tang (YXWST), Si-Wu Tang (SWT) combined with Erzhi Wan (EZW; SWT + EZW), Xiaofeng–Zhiyang Particles (XFZYP), Jingfu–Zhiyang Particles (JFZYP), Baifuzhi–Weiliang Tang (BFWLT), SWT, Chou’s self-made decoction (CSMD), Mahuang–Lianqiao–Chixiaodou Tang (MLCT) combined with Yiyifuzhi–Baijiang Tang (YFBT; MLCT + YFBT), Qufeng decoction (QFD), Xiao–Yang–Ke–Li (XYKL), and Feng–Xueqing–Yin (FXQY). The components of all CHM formulas are listed in Supplementary Table S2.

**TABLE 1:** Characteristics of selected studies.

Study (year)	CHM Intervention	No. of patients (I/C)	Age (years)	Dosage and frequency	Duration	Overall effective rate	Pruritus severity assessment	Pruritus score (before→after)
Zhu et al. (2004)	Yangxue Wensheng Decoction	17/15	I: 70.2 (4.3) C: 71.6 (3.1)	NA, 2 times/d	2 M	I: 16/17 C: 14/15	NA	NA
He, (2006)	Siwu Tang + Erzhi Wan	20/18	I: 50.5 C: 49.8	NA, 2 times/d	1 Week	I: 19/20 C: 11/18	NA	NA
(Wang, (2013)	Xiaoyang Particles	21/22	NA	1 pack, 2 times/d	2 M	NA	VAS	I: 8.08 (1.02)→ 5.05 (2.03) C: 7.89 (1.32)→ 8.03 (1.42)
Wang et al. (2015)	Touxie-Jiedu-Zhiyang Formula	39/39	I: 49 (8) C: 52 (10)	100 ml 2 times/d	3 M	I: 35/39 C: 27/39	VAS	I: 6.95 (1.47)→ 2.31 (1.28) C: 6.87 (1.53)→ 2.94 (1.35)
Yu et al. (2017)	UCG	65/63	I: 35-68 C: 36-70	5 g, 4 times/d	3 M	I: 49/65 C: 36/63	VAS	I: 8.17 (1.94) → 4.28 (1.45) C: 8.21 (1.78) → 6.45 (1.91)
Diao et al. (2018)	Touxie-Jiedu-Zhiyang Decoction	25/25	I: 62.3 (4.8) C: 61.6 (5.4)	NA, 2 times/d	3 M	I: 23/25 C: 15/25	TCM new drug clinical research guideline	I: 2.51 (.79)→ .72 (.34)
Ge. (2018)	Xiaofeng Zhiyang Particles	45/45	I: 56.03 (7.26) C: 55.24 (7.31)	3 packs 2 times/d	0.5 M	I: 41/45 C: 33/45	TCM new drug clinical research guideline	I: 102.37 (16.87)→ 40.32 (20.16) C: 99.26 (17.45)→ 64.21 (25.02)
Cao, (2019)	UCG	40/40	I: 59.7 C: 59.8	5 g, 4 times/d	2 M	I: 38/40 C: 32/40	NRS	I: 7.89 (1.31)→3.10 (.93) C: 7.95 (1.43)→5.37 (1.02)
Kun et al. (2019)	UCG	23/23	I:45.3 (5.3) C:46.1 (4.9)	5 g, 4 times/d	3 M	I: 21/23 C: 16/23	VAS	I: 8.13 (1.77) → 4.73 (1.41) C: 8.40 (2.07) → 6.28 (2.19)
Kuo et al. (2019)	UCG	30/30	I: 42.6 (3.2) C: 41.9 (3.4)	5 g, 4 times/d	3 M	I: 27/30 C: 19/30	NA	NA
(Li and Guo, 2019)	UCG	52/50	I: 47.2 (3.7) C: 46.7 (4.2)	5 g, 4 times/d	3 M	I: 39/52 C: 28/50	VAS	I: 8.18 (1.69) → 4.31 (1.52) C: 8.20 (1.96) → 6.38 (1.88)
Liu et al. (2019)	Jingfu Zhiyang Particles	51/51	I: 55.43 (11.02) C: 55.47 (11.01)	6 g, 3 times/d	1 M	I: 48/51 C: 39/51	Self-made PS questionnaire	I: 16.26 (4.49)→ 6.01 (3.54) C: 16.33 (4.51)→ 9.73 (3.55)
Shi, (2019)	Touxie-Jiedu-Zhiyang Decoction	20/20	I: 45.24 (2.78) C: 45.21 (2.42)	NA, 2 times/d	3 M	I: 20/20 C: 16/20	NA	NA
Chen, (2020)	Touxie-Jiedu-Zhiyang Decoction	30/30	I: 56.13 (7.45) C: 56.34 (7.12)	NA, 2 times/d	3 M	NA	VAS	I: 7.35 (2.13)→ 2.96 (1.22) C: 7.32 (2.24)→ 7.34 (2.37)
Chen et al. (2020)	UCG	50/50	I: 65.72 (10.33) C: 64.12 (10.54)	5 g, 4 times/d	3 M	I: 42/50 C: 33/50	VAS	I: 7.62 (1.02)→3.36 (1.06) C: 7.54 (.98)→ 5.53 (1.78)
							5-D Itch Scale	I: 17.37 (3.56)→ 6.44 (1.59) C: 16.98 (3.72)→ 10.82 (2.31)
							DLQI	I: 21.84 (5.53)→ 8.36 (2.21) C: 21.54 (5.70)→10.55 (3.88)
Fan, (2020)	Fan’s Touxie-Jiedu-Zhiyang Decoction	47/47	I: 27.32 (2.13) C: 27.37 (2.42)	500 ml 2 times/d	NA	NA	VAS	I: 7.31 (2.11)→ 2.86 (1.08) C: 7.17 (2.16)→ 5.46 (1.75)
Yang and Yang, (2020)	Baifuzhi Weiliang Decoction	29/30	I: 49.1 (8.5) C: 49.5 (8.2)	150 ml, 2 times/d	3 M	I: 28/29 C: 26/30	Sergio PS	I: 30.9 (8.8)→ 4.3 (1.9) C: 30.4 (8.6)→ 10.8 (2.5)
Zhao, (2020)	Siwu Decoction	30/30	I: 61.17 (13.35) C: 58.83 (14.61)	NA, 2 times/d	1 M	I: 16/30 C: 8/30	VAS	I: 6.23 (1.22)→ 3.33 (1.42) C: 6.23 (1.63)→ 4.47 (1.20)
Zhou, (2021)	Chou’s Self-made Decoction	21/21	I: 43.59 (3.72) C: 43.46 (3.68)	150 ml, 2 times/d	2 M	I: 19/21 C: 16/21	NA	NA
Dou, (2021)	Modified Yangxue Runfu Yin	40/40	I: 53.56 (15.67) C: 53.62 (15.48)	100 ml 2 times/d	0.5 M	I: 39/40 C: 32/40	VAS	I: 8.02 (2.26)→ 3.88 (1.84) C: 7.74 (1.54)→ 5.41 (2.61)
							Duo PS	I: 31.21 (8.91)→ 16.11 (2.21) C: 28.91 (9.21)→ 24.81 (7.91)
Jin, (2021)	Mahuang Lianqiao Chixiaodou + Yiyifuzhi baijiang Decoction	30/30	I: 53.26 (11.38) C: 53.26 (11.38)	100 ml, 2 times/d	6 M	I: 23/30 C: 17/30	Sergio PS	NA
Li. (2021)	UCG	50/50	I: 51.21 (1.92) C: 49.39 (2.74)	5 g, 4 times/d	14 Weeks	I: 41/50 C: 29/50	NA	I: 8.29 (1.70)→ 4.42 (1.63) C: 8.31 (2.07)→ 6.49 (1.99)
Wang, (2021)	Feng Xueqing Yin	16/16	I: 57.19 (5.79) C: 53.50 (9.14)	1 pack,2 times/d	1 M	NA	VAS	I: 39.21 (2.50)→ 17.08 (3.05) C: 39.91 (2.76)→ 29.06 (2.86)
Wu and Gao, (2021)	Modified Qufeng Decoction	36/35	I: 45.8 (8.4) C: 46.3 (8.6)	100 ml, 2 times/d	1 M	I: 34/36 C: 23/35	VAS	I: 27.65 (3.24)→ 4.18 (1.20) C: 27.49 (3.20)→ 12.84 (3.62)
Xi. (2021)	UCG	58/58	I: 47.88 (3.52) C: 47.79 (3.41)	5 g, 4 times/d	NA	I: 56/58 C: 45/58	VAS	I: 7.21 (1.72)→ 1.47 (.34) C: 7.23 (1.71)→ 3.48 (.53)

CT, conventional treatment; C, control group; DLQI, dermatology life quality index; HD, hemodialysis; HP, hemoperfusion; I, intervention group; M, month; NA, not applicable; NRS, numeric rating scale; PS, pruritis score; TCM, traditional Chinese medicine; UCG, uremic clearance granule; VAS, visual analogue scale.

a Conventional treatment (Acid-base status with electrolyte balanced, sodium and fluid restriction, blood pressure maintenance) for chronic kidney disease in both Intervention and control group.

b Chinese medicine including: Huangqi, Danggui, Danshen, Baishao, Baizhu, Difuzi, Baixianpi, Chuanxiong, Tufuling, Jingjie, Fangfeng, Dahuang.

### Risk of bias assessment

The risk of bias assessment summary and graph of included studies are shown in Supplementary Figures S1a, b. The domain of deviations from the intended interventions and missing outcome data showed a low risk of bias. However, the domain of the overall bias and outcome measurement manifested a high risk of bias due to subjective outcomes, such as self-assessed pruritic scores. In randomization process domain, most studies had unclear risks of bias resulting from concerns about allocation concealment. Only one study (Zhu et al., 2004) showed a high risk of bias for no statistical data, which could cause baseline imbalance. In the reported result domain, most studies showed a low risk of bias, but one study (Yang and Yang, 2020) scored high for risk of bias because of incomplete outcome data.

#### Primary outcome: Visual analog scale

**FIGURE 2:**
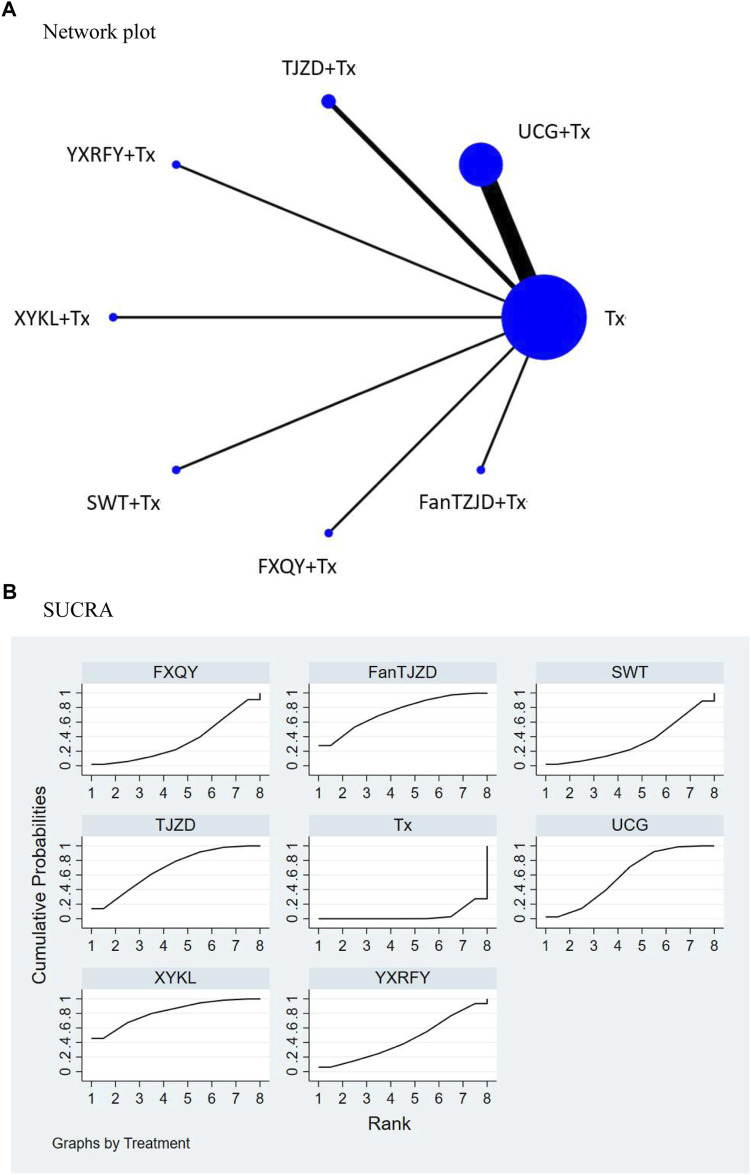
Results of network meta-analysis for the visual analog scale (VAS). **(A)** Network plot: Each node corresponds to one treatment, node size is proportional to the number of subjects, and line thickness is proportional to the number of randomized controlled trials providing comparison data. **(B)** SUCRA: Surface under the cumulative ranking curve. SUCRA determines the overall ranking of each treatment. A larger area under the curve corresponds to a higher ranking. Higher ranking indicates a better treatment in the network meta-analysis. Abbreviations: *FanTJZD,* Fan’s Touxie Jiedu Zhiyang Decoction; *FXQY,* Feng Xueqing Yin; *SWT*, Siwu Tang; *TJZD*, Touxie Jiedu Zhiyang Decoction; *Tx*, Conventional treatment; *UCG*, Uremic Clearance Granules; *XYKL*, Xiaoyang Ke Li; *YXRFY*, Yangxue Runfu Yin.

VAS scores were reported in 14 studies including seven types of CHM formulas in the network meta-analysis. Seven studies involved UCG, two involved TJZD, and the other five involved different formulas including FanTZJD, YXRFY, XYKL, SWT, and FXQY. The network plot (Figure 2A) shows that UCG publications involved the highest number of subjects, and FXQY publications involved the lowest number of subjects. Most trials reported the comparison between Tx and UCG + Tx. The netleague table (Table 2) demonstrates direct and indirect comparisons of CHM formulas’ effectiveness in reducing VAS scores. Compared with Tx, XYKL + Tx, FanTJZD + Tx, TJZD + Tx, and UCG + Tx significantly reduced the VAS scores of UP in dialysis patients. XYKL + Tx (Mean difference (MD), −2.98; 95% CI, −5.05 to −.91) was the most effective at reducing the VAS score, followed by FanTJZD + Tx (MD, −2.60; 95% CI, −4.48 to −.72). The SUCRA rankings of the abilities of the treatments to reduce VAS scores of UP in dialysis patients (Figure 2B; Table 3) was as follows (in descending order): XYKL (SUCRA = 81.7), FanTJZD (SUCRA = 74.0), TJZD (SUCRA = 68.8), UCG (SUCRA = 59.7), YXRFY (SUCRA = 44.1), FXQY (SUCRA = 34.1), SWT (SUCRA = 33.3), and Tx (SUCRA = 4.3).

**TABLE 2:** Netleague table comparing different Chinese herbal medicine treatments in terms of visual analog scale.

XYKL + Tx	.38 (−2.42,3.18)	.61 (−1.88,3.11)	.92 (−1.27,3.11)	1.45 (−1.46,4.36)	1.78 (−.96,4.52)	1.84 (−.98,4.66)	2.98 (.91,5.05)*
−.38 (−3.18,2.42)	FanTJZD + Tx	.23 (−2.10,2.57)	.54 (−1.47,2.55)	1.07 (−1.71,3.85)	1.40 (−1.20,4.00)	1.46 (−1.22,4.14)	2.60 (.72,4.48)*
−.61 (−3.11,1.88)	−.23 (−2.57,2.10)	TJZD + Tx	.31 (−1.25,1.87)	.84 (−1.63,3.30)	1.17 (−1.10,3.43)	1.23 (−1.13,3.58)	2.37 (.98,3.75)*
−.92 (−3.11,1.27)	−.54 (−2.55,1.47)	−.31 (−1.87,1.25)	UCG + Tx	.53 (−1.64,2.69)	.86 (−1.08,2.79)	.92 (−1.12,2.95)	2.06 (1.34,2.77)*
−1.45 (−4.36,1.46)	−1.07 (−3.85,1.71)	−.84 (−3.30,1.63)	−.53 (−2.69,1.64)	YXRFY + Tx	.33 (−2.39,3.05)	.39 (−2.40,3.18)	1.53 (−.51,3.57)
−1.78 (−4.52,0.96)	−1.40 (−4.00,1.20)	−1.17 (−3.43,1.10)	−.86 (−2.79,1.08)	−.33 (−3.05,2.39)	FXQY + Tx	.06 (−2.56,2.68)	1.20 (−.60,3.00)
−1.84 (−4.66,0.98)	−1.46 (−4.14,1.22)	−1.23 (−3.58,1.13)	−.92 (−2.95,1.12)	−.39 (−3.18,2.40)	−.06 (−2.68,2.56)	SWT + Tx	1.14 (−.77,3.05)
−2.98 (−5.05,-.91)*	−2.60 (−4.48,-.72)*	−2.37 (−3.75,-.98)*	−2.06 (−2.77,-1.34)*	−1.53 (−3.57,0.51)	−1.20 (−3.00,0.60)	−1.14 (−3.05,0.77)	**Tx**

Relative risks and mean differences with 95% confidence intervals are presented. Columns versus row should be read from left to right and are ordered by decreasing treatment efficacy. The intervention in the top left shows the best outcome measurement in the network meta-analysis. Light yellow boxes (*) represent statistically significant comparisons.

**TABLE 3:** SUCRA ranking of visual analog scale in Chinese herbal medicine treatment in dialysis patients.

Treatment	SUCRA	Pr best	Mean rank
Tx	4.3	0.0	7.7
UCG	59.7	2.5	3.8
TJZD	68.8	13.7	3.2
YXRFY	44.1	6.1	4.9
XYKL	81.7	45.7	2.3
SWT	33.3	2.2	5.7
FXQY	34.1	2.0	5.6
Fan’s TJZD	74.0	27.8	2.8

#### Secondary outcome: Overall effective rate

The overall effective rate (ER) was reported in 21 studies including 12 types of CHM formulas in the network meta-analysis. Eight studies involved UCGs, three involved TJZD, and the other 10 involved the following different formulas: YXRFY, YXWST, SWT + EZW, XFZYP, JFZYP, BFWLT, SWT, CSMD, MLCT + YFBT, and QFD. For different CHM interventions, the network plot (Supplementary Figure S3a) shows that the highest number of publications and subjects involved UCG treatment, whereas YXWST had the fewest subjects and publications. Most trials reported comparisons between conventional treatment (Tx) and UCG + Tx. The netleague table (Supplementary Figure S3a) demonstrates direct and indirect comparisons of CHM formulas that increased ER. Compared with Tx, SWT + Tx, QFD + Tx, TJZD + Tx, UCG + Tx, XFZYP + Tx, JFZYP + Tx, and YXRFY + Tx significantly increased the ER of UP treatment in dialysis patients. SWT + Tx (RR, 2.00; 95% CI, 1.01–3.95) treatment increased the ER most effectively, followed by SWT + EZW + Tx (RR, 1.55; 95% CI, 1.06–2.28). SWT + EZW + Tx, QFD + Tx, TJZD + Tx, and UCG + Tx were superior to YXRFY + Tx (Supplementary Figure S3a). The surface under the cumulative ranking curve (SUCRA) shows the overall rank of each treatment. The SUCRA rankings of the ER of CHM treatments for UP in dialysis patients in descending order (Supplementary Figure S3b; Supplementary Table S4a) was as follows: SWT (SUCRA = 89.5), SWT + EZW (SUCRA = 79.3), QFD (SUCRA = 75.4), TJZD (SUCRA = 62.9), MLCT + YFBT (SUCRA = 61.8), UCG (SUCRA = 55.7), XFZYP (SUCRA = 49.3), JFZYP (SUCRA = 47.5), YXRFY (SUCRA = 44.8), CSMD (SUCRA = 40.3), BFWLT (SUCRA = 26.3), YXWST (SUCRA = 10.5), and Tx (SUCRA = 6.6).

#### Secondary outcome: C-reactive protein

CRP levels were reported in eight studies including five types of CHM formulas in the network meta-analysis. Four studies involved UCG, and the other four involved different formulas including FanTJZD, SWT, XYKL, and YXRFY. Of the different CHM interventions, the network plot (Supplementary Figure S4a) shows that publications on UCG involved the highest numbers of subjects and those on SWT involved the lowest numbers. Most trials reported comparisons between Tx and UCG + Tx. The netleague table (Supplementary Table S3b) demonstrates direct and indirect comparisons of the effectiveness of the CHM formulas in reducing CRP levels. Compared with Tx, XYKL + Tx and UCG + Tx significantly reduced CRP levels in dialysis patients. XYKL + Tx (MD, −5.01; 95% CI, −7.27 to −2.75) was superior to all other treatments in its reduction of CRP levels followed by UCG + Tx (MD, −1.87; 95% CI, −2.29 to −.76). The SUCRA rankings of the CHMs’ effectiveness at reducing CRP levels in dialysis patients with UP (Supplementary Figure S4b; Supplementary Table S4b) was as follows, in descending order: XYKL (SUCRA = 99.0), UCG (SUCRA = 59.5), YXRFY (SUCRA = 55.8), FanTJZD (SUCRA = 39.8), SWT (SUCRA = 38.8), and Tx (SUCRA = 7.1).

#### Secondary outcome: Adverse drug reactions

Adverse drug reactions (ADRs) were reported in five studies including five types of CHM formulas, JFZYP, TJZD, UCG, BFWLT, and SWT, in the network meta-analysis. There was no significant difference in ADRs between Tx and the adjunctive treatments with CHM formulas (Supplementary Figure S5). However, the studies only consisted of JFZYP + Tx, TJZD + Tx, UCG + Tx, BFWLT + Tx, and SWT + Tx treatment arms. No further network meta-analysis was performed with insufficient treatment arms.

### Cluster ranking plot of different CHM treatments for UP in dialysis patients

**FIGURE 3:**
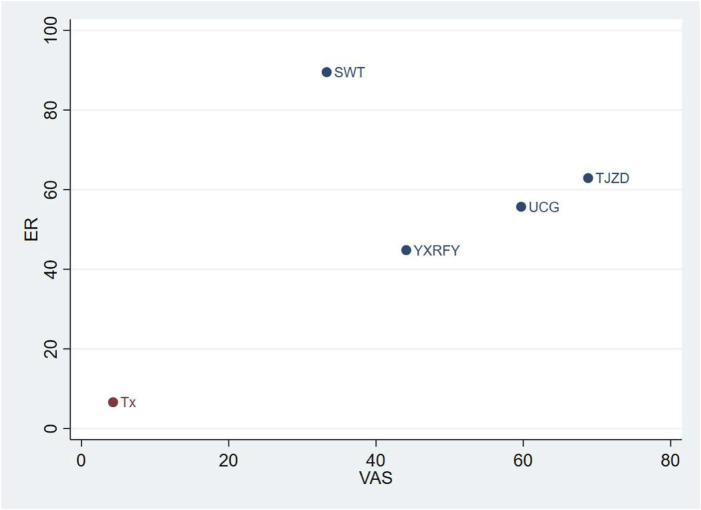
Cluster ranking plot. The cluster ranking plot is based on surface under the cumulative ranking curve (SUCRA) values. The vertical column indicates SUCRA values for the overall effective rate (ER); the horizontal column indicates SUCRA values for the visual analog scale (VAS). CHM treatments in the upper right corner improve the overall effective rate and visual analog scale more than the other treatments.

The cluster ranking plot was based on predicted SUCRA values for ER and VAS scores (Figure 3). There were data for both ER and VAS scores for the following four CHM formulas: SWT, TZJD, UCG, and YXRFY. TJZD + Tx was predicted to be more effective than UCG + Tx and YXRFY + Tx in improving both ER and VAS scores. SWT + Tx was the most effective treatment for improving the ER but was the least effective for reducing VAS scores.

### Publication bias

Funnel plots were conducted to detect publication bias of studies reporting ER and VAS scores (Supplementary Figure S2). Both funnel plots showed asymmetrical distributions, indicating potential publication bias in the included studies.

## Discussion

This network meta-analysis included 25 studies comprising 1,703 dialysis patients with UP. Seven adjunctive CHM treatments showed significantly higher efficacy than single Tx including SWT, QFD, TJZD, UCG, XFZYP, JFZYP, and YXRFY. SWT + Tx was the most effective CHM intervention in improving the ER. Compared with Tx alone, four adjunctive CHM treatments significantly reduced the VAS scores of UP, including XYKL, FanTJZD, TJZD, and UCG. In addition, two treatments, XYKL and UCG, significantly reduced CRP levels compared with Tx. XYKL + Tx was the most effective CHM intervention for reducing both VAS scores and CRP levels. Considering both ER and VAS scores, TJZD + Tx was the most effective treatment for alleviating UP for dialysis patients, followed by UCG + Tx. Compared with Tx alone, no significant increased risk of ADRs was found when it was combined with adjunctive CHM treatments.

Previous reviews have reported the effectiveness of CHM treatments for pruritus. CHM herbal bath therapy, which contains 11 Chinese medicinal herbs, could be an effective complementary treatment for alleviating UP (Xue et al., 2019). Lu et al. (Lu et al., 2021) conducted a systematic review and showed that UCG reduced VAS scores and inflammatory markers, including hsCRP, TNF-α, β2-MG, and IL-6. In addition, the herb *Fumaria parviflora* was found to decrease VAS and Duo scores in dialysis patients with UP in a RCT (Akrami, 2017). Another RCT showed that Xiao Feng San, a traditional CHM for attenuating pruritus, decreased Duo scores of UP (Wang et al., 2010). Bai et al. (Akrami, 2017) conducted a RCT to evaluate the efficacy of topical Chinese herb-based emollient Lifu paste on dialysis patients with UP; Lifu paste application significantly decreased VAS scores of UP. CHM, including herbs and herbal baths, may be an effective alternative for attenuating UP in dialysis patients (Lu et al., 2021).

SWT + Tx increased the ER of UP in dialysis patients best. SWT consists of four herbs including *Rehmannia glutinosa* (Shoudihuang), *Angelicae sinensis* (Danggui), *Paeonia lactiflora* (Baishao), and *Ligusticum chuanxiong* (Chuanxiong). An SWT aqueous extract exhibited anti-pruritic and anti-inflammatory effects in mice models. *P. lactiflora* exhibited anti-pruritic effects by inhibiting histamine release from mast cells and attenuating IgE-related itching sensations (Dai et al., 2002; Lee et al., 2008). An extract of *R. glutinosa* was reported to be anti-inflammatory because it inhibited IL-4, TNF-α, VCAM-1, and ICAM-1 in the ear cell of mouse model (Sung et al., 2011). Topical application of *A. sinensis* alleviated dermatitis by reducing cytokines, such as IL-4, IL-6, TNF-α, and IFN-γ (Lee et al., 2016).

*Schizonepeta tenuifolia* (Jingjie), Periostracum Cicadae (Chantui), *Saposhnikovia divaricata* (Fangfeng), *A. sinensis* (Danggui), and *Sophora flavescens* (Kushen) are important herbs in XYKL. In mice, *S. tenuifolia* reduced serum levels of IgE, TNF, and IL-6 and suppressed the NF-κB inflammation pathway (Choi et al., 2013). Components of *S. tenuifolia*, including menthone, isomenthone, pulegone, piperitone, and β-caryophyllene, have anti-inflammatory properties (Bai et al., 2021). Periostracum Cicadae, which is the slough of cicada molting, is commonly used for dermatitis treatment in CHM (Wang, 2013). N-Acetyldopamine dimers, a compound extracted from Periostracum Cicadae, showed anti-inflammatory effects *via* regulation of Th1 and Th17 cells (Thapa et al., 2020). Compounds of *S. divaricata* (Fangfeng) were reported to have anti-inflammatory and immunoregulatory effects (Yang et al., 2020). *A. sinensis* (Danggui) and *S. flavescens* (Kushen) are used to treat inflammatory diseases. Sodium ferulate (SF) and oxymatrine, two ingredients extracted from the radices of *A. sinensis* (Danggui) and *S. flavescens* (Kushen), respectively, reduced CRP and INF-γ in a cell model (Yuan et al., 2011). Methanol extracts of *S. flavescens* (Kushen) attenuated 5-HT-induced scratching in mice in a dose-dependent manner (Yamaguchi-Miyamoto et al., 2003). Topical application of *A. sinensis* (Danggui) significantly suppressed the scratching behavior (Lee et al., 2016). Anti-inflammatory and anti-pruritic effects have been observed in the aforementioned herbs, which may explain how XYKL reduced the VAS scores and CRP levels of dialysis patients with UP.

TZJD was the most effective at improving the ER and reducing VAS scores based on the cluster ranking plot. *Astragalus membranaceus* (Huangqi), a significant ingredient of TZJD, improved atopic dermatitis by significantly decreasing an inflammatory biomarker (TNF-α) and Th2 cytokine levels (Kim et al., 2013). XYKL and TZJD both contain *A. sinensis* (Danggui), which has commonly been used for treating cutaneous pruritus. A previous study showed that a CHM formula containing a mixture of *A. membranaceus* (Huangqi) and *A. sinensis* (Danggui) exhibited anti-allergic and anti-inflammatory effects by reducing levels of cytokines (IL-4, IL-6, IFN-γ, TNF-α, and IL-1β) and inflammatory mediators (Choi et al., 2016).

In this network meta-analysis, we provided direct and indirect comparisons between different CHM formulas as UP interventions, which will provide clinical suggestions for use of different CHM formulas by clinicians as adjunctive treatments for UP in dialysis patients. However, our study had limitations. First, a single-trial inclusion of several CHM formulas could result in small sample sizes, which could lead to inconsistent outcomes. For instance, compared with Tx, SWT + Tx showed a significantly higher ER but did not significantly reduce VAS scores. Second, the included trials were from a single country, potentially limiting the generalizability of the results. Third, variations in CHM dosages and treatment courses (from 2 weeks to over 3 months) could have affected the outcome measures. Forth, minor improvement of VAS manifests limited role of CHM in relieving uremic pruritus in dialysis patients. Last, about thirty percent of UP patients benefits from placebo effect while comparing to Difelikefalin (Fugal and Serpa, 2022). However, lack of placebo comparison in control groups from included RCTs in our study might affect the result of effectiveness.

## Conclusion

SWT + Tx was superior to other CHM interventions in increasing ER. XYKL + Tx was the most effective CHM intervention in reducing both VAS scores and CRP levels. TZJD + Tx was the most effective intervention, and significantly improved both the ER and VAS scores, followed by UCG + Tx. No obvious ADRs were noted after CHM treatment administration. Head-to-head comparisons may aid in shared decision-making and provide different adjunctive CHM treatment options for UP in dialysis patients.

## Data Availability

The datasets presented in this study can be found in online repositories. The names of the repository/repositories and accession number(s) can be found in the article/Supplementary Material.
