# Supplementary material for: Comparative efficacy of Chinese herbal medicines for dialysis patients with uremic pruritus: A systematic review and network meta-analysis
Source: Front Pharmacol. 2023 Jan 17;14:1064926. doi: 10.3389/fphar.2023.1064926 (PMC9886678; doi:10.3389/fphar.2023.1064926)
Supplement: Supplementary file 1 [file DataSheet1.docx]

Supplementary Material

# Supplementary Table 1. Search strategy

| PUBMED |
| --- |
| 1. Chronic kidney disease OR kidney injury OR kidney failure OR chronic renal failure OR end-stage renal disease OR end stage renal disease OR dialysis OR hemodialysis OR peritoneal dialysis 2. Uremic OR Uremia OR uremias 3. #1 OR #2 4. Pruritus OR Pruritis OR itch* OR xerosis OR skin problems OR skin disorders 5. Chinese patent medicine OR Chinese patent drugs OR Medicine, Chinese Traditional OR chinese herbologia OR Chinese medicine OR Chinese material medical OR Chinese herbs OR Chinese herbal medicine OR Herbal Medicine OR Chin Tradit Pat Med 6. Formula 7. (Decoction OR Tang) 8. (Pill OR Wan) 9. San [Title/Abstract] 10. Granule 11. Pian 12. Powder 13. #3 AND #4 AND (#5 OR #6 OR #7 OR #8 OR #9 OR #10 OR #11 OR #12) |
| EMBASE |
| 1. 'chronic kidney disease'/exp OR 'chronic kidney disease' OR (chronic AND ('kidney'/exp OR kidney) AND ('disease'/exp OR disease)) OR 'kidney injury'/exp OR 'kidney injury' OR (('kidney'/exp OR kidney) AND ('injury'/exp OR injury)) OR 'kidney failure'/exp OR 'kidney failure' OR (('kidney'/exp OR kidney) AND ('failure'/exp OR failure)) OR 'chronic renal failure'/exp OR 'chronic renal failure' OR (chronic AND ('renal'/exp OR renal) AND ('failure'/exp OR failure)) OR 'end-stage renal disease'/exp OR 'end-stage renal disease' OR ('end stage' AND ('renal'/exp OR renal) AND ('disease'/exp OR disease)) OR 'end stage renal disease'/exp OR 'end stage renal disease' OR (end AND stage AND ('renal'/exp OR renal) AND ('disease'/exp OR disease)) OR 'dialysis'/exp OR dialysis OR 'hemodialysis'/exp OR hemodialysis OR 'peritoneal dialysis'/exp OR 'peritoneal dialysis' OR (peritoneal AND ('dialysis'/exp OR dialysis)) 2. uremic OR 'uremia'/exp OR uremia OR uremias 3. #1 OR #2 4. 'pruritus'/exp OR pruritus OR 'pruritis'/exp OR pruritis OR itch* OR 'xerosis'/exp OR xerosis OR 'skin problems' OR (('skin'/exp OR skin) AND problems) OR 'skin disorders' OR (('skin'/exp OR skin) AND ('disorders'/exp OR disorders)) 5. 'chinese medicine'/exp OR 'chinese medicine' OR 'herbal medicine'/exp OR 'herbal medicine' OR 'chinese patent medicine':ti,ab,kw OR 'chinese patent drugs':ti,ab,kw OR 'chinese herbologia':ti,ab,kw OR 'medicine, chinese traditional':ti,ab,kw OR 'chinese material medical':ti,ab,kw OR 'chinese herbs':ti,ab,kw OR 'chinese herbal medicine':ti,ab,kw OR 'chin tradit pat med':ti,ab,kw 6. ('formula'/exp OR formula) 7. ('decoction'/exp OR decoction OR tang) 8. ('pill'/exp OR pill OR wan) 9. san:ti,ab,kw 10. ('granule'/exp OR granule) 11. ('pian'/exp OR pian) 12. ('powder'/exp OR powder) 13. #3 AND #4 AND (#5 OR #6 OR #7 OR #8 OR #9 OR #10 OR #11 OR #12) |
| Cumulative Index to Nursing and Allied Health Literature (CINAHL) |
| 1. (chronic kidney disease OR kidney injury OR kidney failure OR chronic renal failure OR end-stage renal disease OR end stage renal disease OR dialysis OR hemodialysis OR peritoneal dialysis) 2. (uremic OR Uremia OR uremias) 3. #1 OR #2 4. (Pruritus OR Pruritis OR itch* OR xerosis OR skin problems OR skin disorders) 5. (Chinese patent medicine OR Chinese patent drugs OR traditional Chinese medicine OR chinese herbologia OR Chinese medicine OR Chinese material medical OR Chinese herbs OR Chinese herbal medicine OR herbal medicine OR Chin Tradit Pat Med) 6. Formula 7. (Decoction OR Tang) 8. (Pill OR Wan) 9. Granule 10. Pian 11. Powder 12. San 13. #3 AND #4 AND (#5 OR #6 OR #7 OR #8 OR #9 OR #10 OR #11 OR #12) |
| Cochrane Central Register of Controlled Trials |
| 1. (chronic kidney disease OR kidney injury OR kidney failure OR chronic renal failure OR end-stage renal disease OR end stage renal disease OR dialysis OR hemodialysis OR peritoneal dialysis) 2. (uremic OR Uremia OR uremias) 3. #1 OR #2 4. (Pruritus OR Pruritis OR itch* OR xerosis OR skin problems OR skin disorders) 5. (Chinese patent medicine OR Chinese patent drugs OR traditional Chinese medicine OR chinese herbologia OR Chinese medicine OR Chinese material medical OR Chinese herbs OR Chinese herbal medicine OR herbal medicine OR Chin Tradit Pat Med) 6. Formula 7. (Decoction OR Tang) 8. (Pill OR Wan) 9. Granule 10. Pian 11. Powder 12. San 13. #3 AND #4 AND (#5 OR #6 OR #7 OR #8 OR #9 OR #10 OR #11 OR #12) |
| Airiti library |
| 1. Chronic kidney disease OR kidney failure OR dialysis OR hemodialysis OR peritoneal dialysis OR renal failure OR renal insufficiency OR membranous glomerulonephritis OR nephritis OR uremic OR Uremia OR uremias 2. Pruritus OR Pruritis OR itch* OR xerosis OR Skin Diseases 3. Chinese medicine OR chinese herbal medicine OR chinese and western medicine OR prescription OR (Decoction OR Tang) OR (Pill OR Wan) OR san OR formula OR powder OR granule OR pian OR jian 4. #1 AND #2 AND #3 |
| China National Knowledge Infrastructure |
| 1. 'chronic kidney disease' OR 'kidney failure OR dialysis' OR ' hemodialysis ' OR ' peritoneal dialysis ' OR ' renal failure ' OR ' renal insufficiency ' OR ' membranous glomerulonephritis ' OR ' nephritis ' OR 'uremic' OR 'Uremia' OR 'uremias' 2. 'Pruritus ' OR 'Pruritis ' OR 'itch* ' OR 'xerosis ' OR 'Skin Diseases ' 3. 'Chinese medicine ' OR 'chinese herbal medicine ' OR 'chinese and western medicine ' OR 'prescription ' OR ' (Decoction OR Tang) ' OR ' (Pill OR Wan) ' OR 'san ' OR 'formula ' OR 'powder ' OR 'granule ' OR 'pian ' OR 'jian' 4. #1 AND #2 AND #3 |
| Wangfang |
| 1. 'chronic kidney disease' OR 'kidney failure OR dialysis' OR ' hemodialysis ' OR ' peritoneal dialysis ' OR ' renal failure ' OR ' renal insufficiency ' OR ' membranous glomerulonephritis ' OR ' nephritis ' OR 'uremic' OR 'Uremia' OR 'uremias' 2. 'Pruritus ' OR 'Pruritis ' OR 'itch* ' OR 'xerosis ' OR 'Skin Diseases ' 3. 'Chinese medicine ' OR 'chinese herbal medicine ' OR 'chinese and western medicine ' OR 'prescription ' OR ' (Decoction OR Tang) ' OR ' (Pill OR Wan) ' OR 'san ' OR 'formula ' OR 'powder ' OR 'granule ' OR 'pian ' OR 'jian'   #1 AND #2 AND #3 |

**Supplementary Table 2. Abbreviations and components of Chinese herbal medicine formulas**

| **Abbreviation** | **CHM formula** | **Component** |
| --- | --- | --- |
| BFWLT | Baifuzhi Weiliang Tang | *Typhonii Rhizoma* (Baifuzi), *Atractylodis Rhizoma* (Cangzhu), *Coptis chinensis* (Chuanhuanglian), *Sophora flavescens* (Kushen), *Schizonepetae Herba* (Jingjie), *Saposhnikovia divaricata* (Fangfeng), *Cicadae Periostracum* (Chantui), *Bombyx Batryticatus* (Jiangcan), *Cnidium monnieri* (Shechuangzi), *Xanthium strumarium* (Cangerzi), *Arctii Fructus* (Niubangzi), *Rehmanniae Radix* (Shengdihuang), *Tribulus terrestris* (Jili), *Lonicerae Japonicae Flos* (Jinyinhua) |
| CSMD | Chou's Self-made Decoction | *Astragalus membranaceus* (Huangqi), *Angelicae Sinensis* (Danggui), *Salviae Miltiorrhizae* (Danshen), *Paeonia lactiflora Pall.* (Baishao), *Atractylodes macrocephala* (Baizhu), *Dictamni Cortex.*(Baixianpi), *Kochiae Fructus* (Difuzi), *Ligusticum chuanxiong* (Chuanxiong), *Smilacis Glabrae Rhizoma* (Tufuling), *Schizonepetae Herba* (Jingjie), *Saposhnikovia divaricata* (Fangfeng) |
| EZW | Erzhi Wan | *Fructus Ligustri Lucidi* (Nuzhenzi), *Eclipta prostrata* (Mohanlian) |
| FanTZJD | Fan’s Touxie Jiedu Zhiyang Decoction | *Spatholobi Caulis* (Jixueteng), *Angelicae Sinensis* (Danggui), *Smilacis Glabrae Rhizoma* (Tufuling), *Rehmanniae Radix* (Shengdihuang), *Dioscoreae Rhizoma* (Shanyao), *Clematidis Radix* (Weilingxian), *Polistes mandarinus Saussure* (Fengfang) |
| FXQY | Feng Xueqing Yin | *Rehmanniae Radix* (Shengdihuang), *Angelicae Sinensis* (Danggui), *Paeonia lactiflora Pall.* (Baishao), *Ligusticum chuanxiong* (Chuanxiong), *Scrophularia ningpoensis* (Xuanshen), *Anemarrhena asphodeloides* (Zhimu), *Schizonepetae Herba* (Jingjie), *Saposhnikovia divaricata* (Fangfeng) |
| JFZYP | Jingfu Zhiyang Particles | *Schizonepetae Herba* (Jingjie), *Saposhnikovia divaricata* (Fangfeng), *Chrysanthemum morifolium* (Juhua), *Houttuynia cordata* (Yuxingcao), *Kochiae Fructus* (Difuzi), *Crataegi Fructus* (Shanzha), *Poria cocos* (Fuling) |
| MLCT | Mahuang Lianqiao Chixiaodou Tang | *Ephedra sinica Stapf* (Mahuang), *Forsythia suspensa* (Lianqiao), *Vigna umbellate* (Chixiaodou), *Amygdalus Communis* (Xingren), *Glycyrrhiza uralensis* (Gancao), *Zingiberis Rhizoma Recens* (Shengjiang), *Jujubae Fructus* (Dazao) |
| QFD | Modified Qufeng Decoction | *Polygoni Multiflori Caulis* (Shouwuteng), *Dictamni Cortex.*(Baixianpi), *Spatholobi Caulis* (Jixueteng), *Cicadae Periostracum* (Chantui), *Saposhnikovia divaricata* (Fangfeng), *Glycyrrhiza uralensis* (Gancao), *Uncaria rhynchophylla* (Gouteng), *Tribulus terrestris L.* (Jili), *Kochiae Fructus* (Difuzi), *Sinomenii Caulis* (Qingfengteng), *Cnidium monnieri* (Shechuangzi), *Schizonepetae Herba* (Jingjie), *Paeonia suffruticosa Andr.* (Mudanpi), *Carthami Flos* (Honghua) |
| SWT | Siwu Decoction | *Rehmannia glutinosa* (Shoudihuang), *Angelicae Sinensis* (Danggui), *Paeonia lactiflora Pall.* (Baishao), *Ligusticum chuanxiong* (Chuanxiong) |
| TJZD | Touxie Jiedu Zhiyang Decoction | *Astragalus membranaceus* (Huangqi), *Scutellaria baicalnsis* (Huangqin), *Rehmannia glutinosa* (Dihuang), *Paeonia lactiflora* (Baishao), *Angelicae Sinensis* (Danggui), *Dipsaci Radix* (Xuduan), *Persicae Semen* (Taoren), *Eucommia ulmoides* (Duzhong), *Codonopsis pilosula* (Dangshen), *Chaenomelis Fructus* (Mugua), *Carthami Flos* (Honghua), *Spatholobi Caulis* (Jixueteng), *Clematidis Radix* (Weilingxian), *Angelicae Dahuricae Radix* (Baizhi), *Achyranthis Bidentatae Radix* (Niuxi), *Rheum officinale* (Dahuang), *Glycyrrhiza uralensis* (Gancao) |
| Tx | Conventional treatment | Acid-base status with electrolyte balanced, sodium and fluid restriction, blood pressure maintenance and dialysis with or without anti-pruritic treatment |
| UCG | Uremic clearance granule | *Rheum officinale* (Dahuang), *Bupleurum chinens* (Chaihu), *Astragalus membranaceus* (Huangqi), *Morus alba* (Sangbaipi), *Codonopsis pilosula* (Dangshen), *Paeonia lactiflora* (Baishao), *Ligusticum chuanxiong* (Chuanxiong), *Sophora flavescens* (Kushen), *Chrysanthemum* *morifolium* (Juhua), *Pinellia* *ternata* (Banxia), *Atractylodes* *macrocephala* (Baizhu), *Poria* *cocos* (Fuling), *Polygonum* *multiflorum* (Heshouwu), *Salviae* *Miltiorrhizae* (Danshen), *Herba* *Plantaginis* (Cheqiancao), *Glycyrrhiza* *uralensis* (Gancao) |
| XFZYP | Xiaofeng Zhiyang particle | *Schizonepetae* *Herba* (Jingjie), *Saposhnikovia* *divaricata* (Fangfeng), *Cicadae* *Periostracum* (Chantui), *Angelicae* *Sinensis* (Danggui), *Rehmanniae* *Radix* (Shengdihuang), *Lycii* *Cortex* (Digupi), *Atractylodis* *Rhizoma* (Cangzhu), *Gypsum* *Fibrosum* (Shigao), *Akebiae* *Caulis* (Guanmutong), *Glycyrrhiza* *uralensis* (Gancao) |
| XYKL | Xiaoyang Ke Li | *Schizonepetae* *Herba* (Jingjie), *Saposhnikovia* *divaricata* (Fangfeng), *Cicadae* *Periostracum* (Chantui), *Anemarrhena* *asphodeloides* (Zhimu), *Gypsum* *Fibrosum* (Shigao), *Atractylodis* *Rhizoma* (Cangzhu), *Sophora* *flavescens* (Kushen), *Angelicae* *Sinensis* (Danggui), *Pheretima* (Dilong), *Rehmanniae* *Radix* (Shengdihuang), *Cannabis* *Fructus* (Huomaren), *Glycyrrhiza* *uralensis* (Gancao), *Rheum* officinale (Dahuang) |
| YFBT | Yiyifuzhi Baijiang Tang | Coicis *Semen* (Yiyiren), *Aconiti* *Lateralis* *Radix* *Praeparata* (Fuzi), *Patrinia* *villosa* (Baijiang) |
| YXRFY | Yangxue Runfu Yin | *Astragalus* *membranaceus* (Huangqi), *Angelicae* *Sinensis* (Danggui), *Rehmanniae* *Radix* (Dihuang), *Kochia* *scoparia* (Difuzi), *Scutellaria* *baicalnsis* (Huangqin), *Asparagus* *cochinchinensis* (Tianmendong), *Ophiopogon* *japonicus* (Maidong), *Persicae* *Semen* (Taoren), *Carthami* *Flos* (Honghua), *Trichosanthis* *Radix* (Tianhuafen), *Cimicifuga* *dahurica* (Shengma) |
| YXWST | Yangxue Wensheng Tang | *Aconiti* *Lateralis* *Radix* *Praeparata* (Fuzi), *Cinnamomum* *cassia* (Rougui), *Rehmanniae* *Radix* (Shengdihuang), *Polygonum* *multiflorum* (Heshouwu), *Angelicae* *Sinensis* (Danggui), *Ligusticum* *chuanxiong* (Chuanxiong), *Salviae* *Miltiorrhizae* (Danshen), *Paeonia* *anomala* (Chishao), *Carthami* *Flos* (Honghua), *Codonopsis* *pilosula* (Dangshen), *Cuscuta* *chinensis* (Tusizi), *Schizonepetae* *Herba* (Jingjie), *Atractylodis* *Rhizoma* (Cangzhu), *Phellodendron* *amurense* (Huangbai), *Glycyrrhiza* *uralensis* (Gancao) |

**Supplementary Table 3. Netleague table comparing different Chinese herbal medicine treatments in terms of overall effective rate** **and C-reactive protein (Secondary outcome)**

1. **Overall effective rate**

| **SWT+Tx** | 0.78 (0.36,1.70) | 0.72 (0.35,1.49) | 0.66 (0.33,1.32) | 0.68 (0.31,1.47) | 0.64 (0.32,1.27) | 0.62 (0.31,1.26) | 0.62 (0.31,1.24) | 0.61 (0.30,1.23) | 0.59 (0.28,1.24) | 0.56 (0.28,1.12) | 0.50 (0.25,1.02) | 0.50 (0.25,0.99)* |
| --- | --- | --- | --- | --- | --- | --- | --- | --- | --- | --- | --- | --- |
| 1.29 (0.59,2.81) | **SWT+**  **EZW**  **+Tx** | 0.92 (0.58,1.46) | 0.85 (0.56,1.28) | 0.87 (0.51,1.48) | 0.82 (0.56,1.21) | 0.80 (0.52,1.23) | 0.79 (0.52,1.20) | 0.78 (0.52,1.19) | 0.76 (0.48,1.22) | 0.72 (0.47,1.08) | 0.65 (0.43,0.99)* | 0.64 (0.44,0.94)* |
| 1.39 (0.67,2.88) | 1.08 (0.68,1.71) | **QFD+Tx** | 0.92 (0.68,1.23) | 0.94 (0.60,1.47) | 0.89 (0.68,1.16) | 0.86 (0.63,1.19) | 0.86 (0.63,1.16) | 0.85 (0.63,1.14) | 0.83 (0.57,1.20) | 0.78 (0.58,1.04) | 0.70 (0.51,0.96)* | 0.70 (0.54,0.90)* |
| 1.52 (0.76,3.05) | 1.18 (0.78,1.78) | 1.09 (0.82,1.46) | **TJZD+Tx** | 1.03 (0.69,1.53) | 0.97 (0.82,1.15) | 0.94 (0.74,1.21) | 0.94 (0.75,1.17) | 0.93 (0.74,1.15) | 0.90 (0.66,1.24) | 0.85 (0.68,1.05) | 0.77 (0.61,0.97)* | 0.76 (0.66,0.88)* |
| 1.48 (0.68,3.21) | 1.15 (0.68,1.96) | 1.06 (0.68,1.66) | 0.97 (0.65,1.45) | **MLCT+**  **YFBT**  **+Tx** | 0.95 (0.65,1.38) | 0.92 (0.60,1.40) | 0.91 (0.61,1.37) | 0.90 (0.60,1.35) | 0.88 (0.55,1.39) | 0.82 (0.55,1.23) | 0.75 (0.49,1.12) | 0.74 (0.51,1.07) |
| 1.56 (0.79,3.11) | 1.22 (0.82,1.80) | 1.12 (0.86,1.46) | 1.03 (0.87,1.22) | 1.06 (0.72,1.54) | **UCG+Tx** | 0.97 (0.78,1.20) | 0.96 (0.80,1.16) | 0.95 (0.80,1.14) | 0.93 (0.70,1.24) | 0.87 (0.73,1.04) | 0.79 (0.65,0.96)* | 0.78 (0.72,0.85)* |
| 1.61 (0.79,3.27) | 1.25 (0.81,1.92) | 1.16 (0.84,1.59) | 1.06 (0.83,1.36) | 1.09 (0.72,1.66) | 1.03 (0.83,1.27) | **XFZYP+Tx** | 0.99 (0.76,1.28) | 0.98 (0.76,1.27) | 0.96 (0.68,1.34) | 0.90 (0.70,1.15) | 0.81 (0.62,1.06) | 0.80 (0.66,0.98)* |
| 1.63 (0.81,3.28) | 1.26 (0.83,1.92) | 1.17 (0.86,1.58) | 1.07 (0.85,1.34) | 1.10 (0.73,1.65) | 1.04 (0.86,1.25) | 1.01 (0.78,1.31) | **JFZYP+Tx** | 0.99 (0.78,1.25) | 0.96 (0.70,1.33) | 0.91 (0.72,1.14) | 0.82 (0.64,1.05) | 0.81 (0.69,0.96)* |
| 1.64 (0.81,3.31) | 1.28 (0.84,1.93) | 1.18 (0.87,1.59) | 1.08 (0.87,1.35) | 1.11 (0.74,1.66) | 1.05 (0.88,1.26) | 1.02 (0.79,1.32) | 1.01 (0.80,1.28) | **YXRFY+Tx** | 0.97 (0.71,1.34) | 0.91 (0.73,1.15) | 0.83 (0.65,1.05) | 0.82 (0.70,0.97)* |
| 1.68 (0.81,3.51) | 1.31 (0.82,2.10) | 1.21 (0.83,1.76) | 1.11 (0.81,1.52) | 1.14 (0.72,1.81) | 1.08 (0.81,1.44) | 1.05 (0.74,1.47) | 1.04 (0.75,1.43) | 1.03 (0.74,1.41) | **CSMD+Tx** | 0.94 (0.68,1.29) | 0.85 (0.61,1.18) | 0.84 (0.64,1.11) |
| 1.80 (0.89,3.61) | 1.40 (0.92,2.11) | 1.29 (0.96,1.74) | 1.18 (0.95,1.47) | 1.21 (0.81,1.81) | 1.15 (0.96,1.37) | 1.12 (0.87,1.44) | 1.10 (0.88,1.39) | 1.09 (0.87,1.37) | 1.07 (0.78,1.46) | **BFWLT+Tx** | 0.91 (0.71,1.15) | 0.90 (0.77,1.05) |
| 1.98 (0.98,4.01) | 1.54 (1.01,2.35)* | 1.43 (1.05,1.94)* | 1.30 (1.03,1.65)* | 1.34 (0.89,2.02) | 1.27 (1.04,1.54)* | 1.23 (0.94,1.61) | 1.22 (0.95,1.56) | 1.21 (0.95,1.54) | 1.18 (0.85,1.64) | 1.10 (0.87,1.40) | **YXWST+Tx** | 0.99 (0.83,1.19) |
| 2.00 (1.01,3.95)* | 1.55 (1.06,2.28)* | 1.44 (1.12,1.85)* | 1.32 (1.13,1.53)* | 1.35 (0.93,1.96) | 1.28 (1.18,1.38)* | 1.24 (1.02,1.52)* | 1.23 (1.04,1.45)* | 1.22 (1.04,1.43)* | 1.19 (0.90,1.57) | 1.11 (0.95,1.30) | 1.01 (0.84,1.21) | **Tx** |

1. **C-reactive protein**

| **XYKL+Tx** | 3.14  (0.62,5.66)* | 3.20  (0.06,6.34)* | 3.85  (0.67,7.03)* | 3.88  (0.82,6.94)* | 5.01  (2.75,7.27)* |
| --- | --- | --- | --- | --- | --- |
| -3.14  (-5.66,-0.62)* | **UCG+Tx** | 0.06  (-2.38,2.51) | 0.71  (-1.78,3.21) | 0.74  (-1.60,3.09) | 1.87  (0.76,2.99)* |
| -3.20  (-6.34,-0.06)* | -0.06  (-2.51,2.38) | **YXRFY+Tx** | 0.65  (-2.47,3.77) | 0.68  (-2.31,3.67) | 1.81  (-0.36,3.98) |
| -3.85  (-7.03,-0.67)* | -0.71  (-3.21,1.78) | -0.65  (-3.77,2.47) | **FanTJZD+Tx** | 0.03  (-3.01,3.07) | 1.16  (-1.07,3.39) |
| -3.88  (-6.94,-0.82)* | -0.74  (-3.09,1.60) | -0.68  (-3.67,2.31) | -0.03  (-3.07,3.01) | **SWT+Tx** | 1.13  (-0.93,3.19) |
| -5.01  (-7.27,-2.75)* | -1.87  (-2.99,-0.76)* | -1.81  (-3.98,0.36) | -1.16  (-3.39,1.07) | -1.13  (-3.19,0.93) | **Tx** |

**Supplementary Table 4. SUCRA ranking of overall effective rate and C-reactive protein (Secondary outcome) in Chinese herbal medicine treatment in dialysis patients**

**a) Overall effective rate**

| **Treatment** | **SUCRA** | **Pr Best** | **Mean Rank** |
| --- | --- | --- | --- |
| Tx | 6.6 | 0.0 | 12.2 |
| UCG | 55.7 | 0.0 | 6.3 |
| TJZD | 62.9 | 0.4 | 5.5 |
| YXRFY | 44.8 | 0.1 | 7.6 |
| YXWST | 10.5 | 0.0 | 11.7 |
| SWT + EZW | 79.3 | 18.0 | 3.5 |
| XFZYP | 49.3 | 0.3 | 7.1 |
| JFZYP | 47.5 | 0.1 | 7.3 |
| BFWLT | 26.3 | 0.0 | 9.8 |
| SWT | 89.5 | 67.8 | 2.3 |
| CSMD | 40.3 | 0.5 | 8.2 |
| MLCT + YFBT | 61.8 | 5.6 | 5.6 |
| QFD | 75.4 | 7.2 | 3.9 |

**b) C-reactive protein**

| **Treatment** | **SUCRA** | **Pr Best** | **Mean Rank** |
| --- | --- | --- | --- |
| Tx | 7.1 | 0.0 | 5.6 |
| XYKL | 99.0 | 95.9 | 1.0 |
| UCG | 59.5 | 0.5 | 3.0 |
| Fan’s TJZD | 39.8 | 0.8 | 4.0 |
| SWT | 38.8 | 0.6 | 4.1 |
| YXRFY | 55.8 | 2.2 | 3.2 |

1. Risk of bias summary


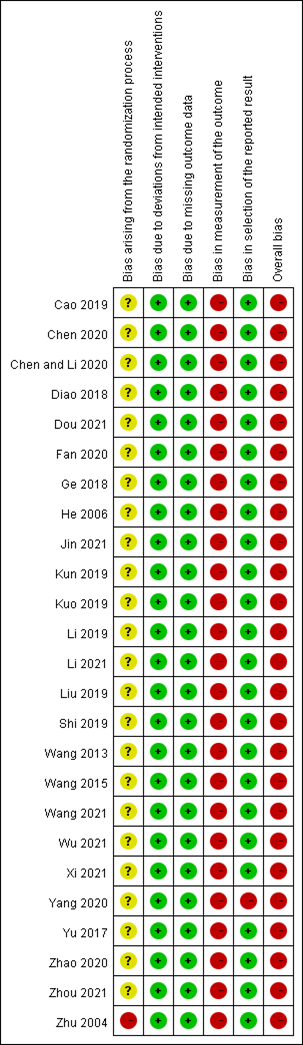


1. Risk of bias graph

**
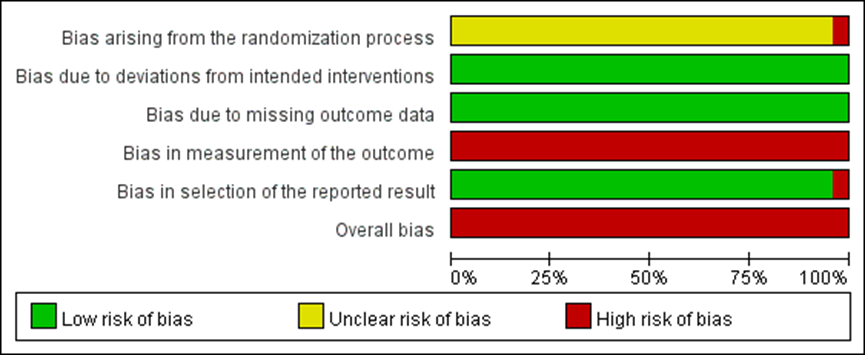
**

**Supplementary Figure 1.** Risk of bias summary and graph of included studies

1. Overall effective rate


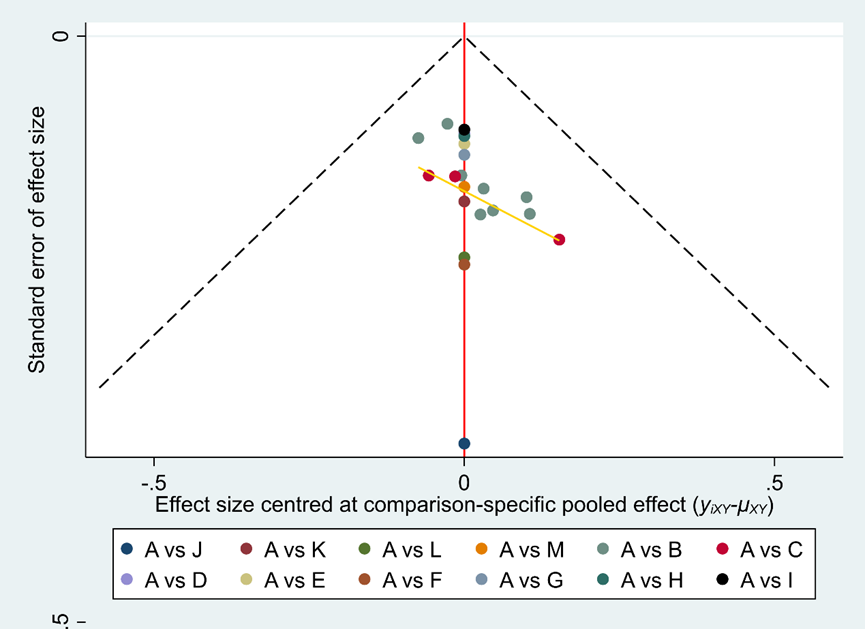


1. Visual analogue scale


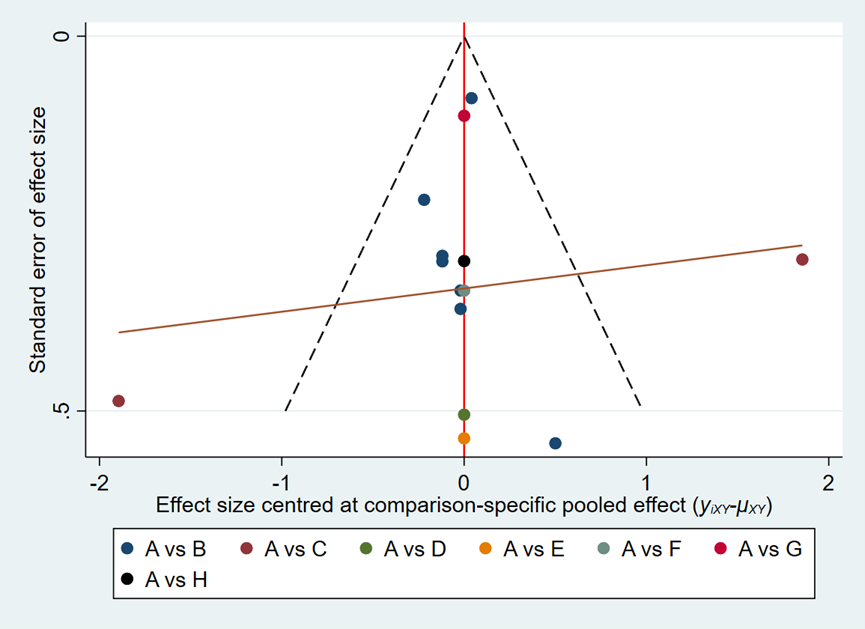


**Supplementary Figure 2.** Funnel plots of different Chinese herbal medicine treatments in overall effective rate and visual analogue scale

Network plot


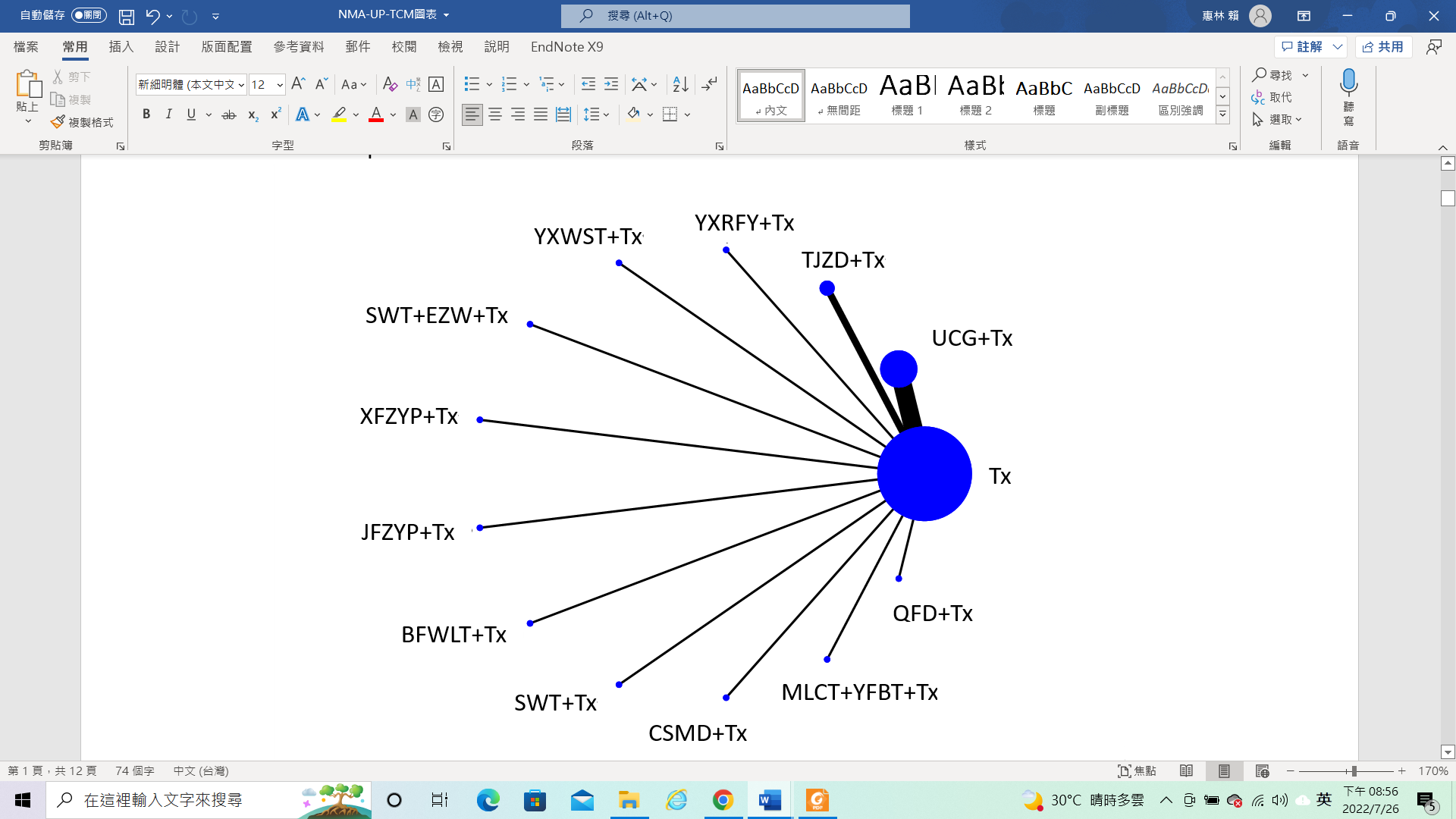


SUCRA


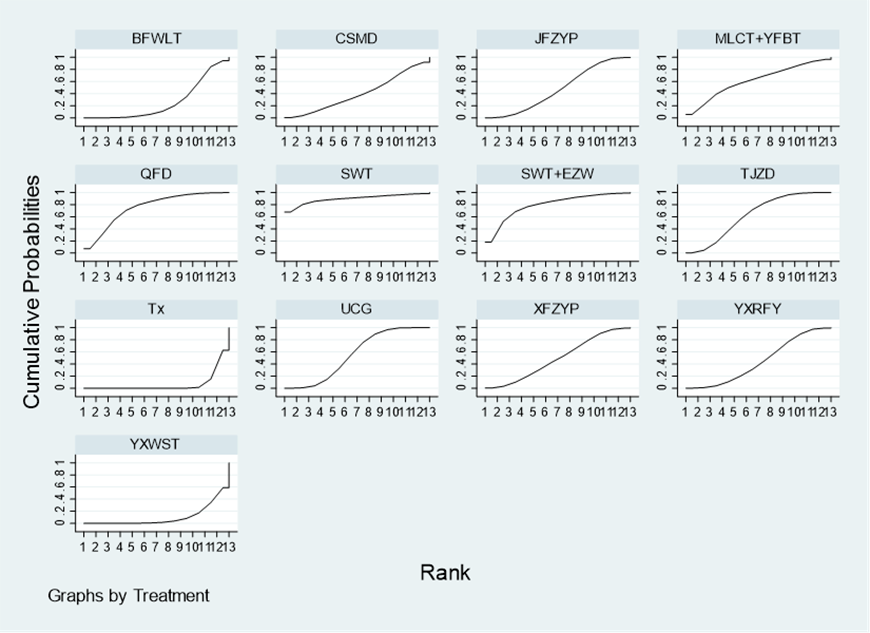


**Supplementary Figure 3. Results of** **Network meta-analysis for overall effectiveness** (a) Network plot: Each node corresponds to one treatment, node size is proportional to the number of subjects, and line thickness is proportional to the number of randomized controlled trials providing comparison data. (b) SUCRA: Surface under the cumulative ranking curve. SUCRA determines the overall ranking of each treatment. A larger area under the curve corresponds to a higher ranking. Higher ranking indicates a better treatment in the network meta-analysis. Abbreviations: *BFWLT*, Baifuzhi Weiliang Tang; *CSMD*, Chou's Self-made Decoction; *EZW*, Erzhi Wan; *JFZYP*, Jingfu Zhiyang Particles; *MLCT*, Mahuang Lianqiao Chixiaodou Tang; *QFD*, Qufeng Decoction; *SWT*, Siwu Tang; *TJZD*, Touxie Jiedu Zhiyang Decoction; *Tx*, Conventional treatment; *UCG*, Uremic Clearance Granules; *XFZYP*, Xiaofeng Zhiyang Particles; *YFBT*, Yiyifuzhi Baijiang Tang; *YXRFY*, Yangxue Runfu Yin; *YXWST*, Yangxue Wensheng Tang

Network plot


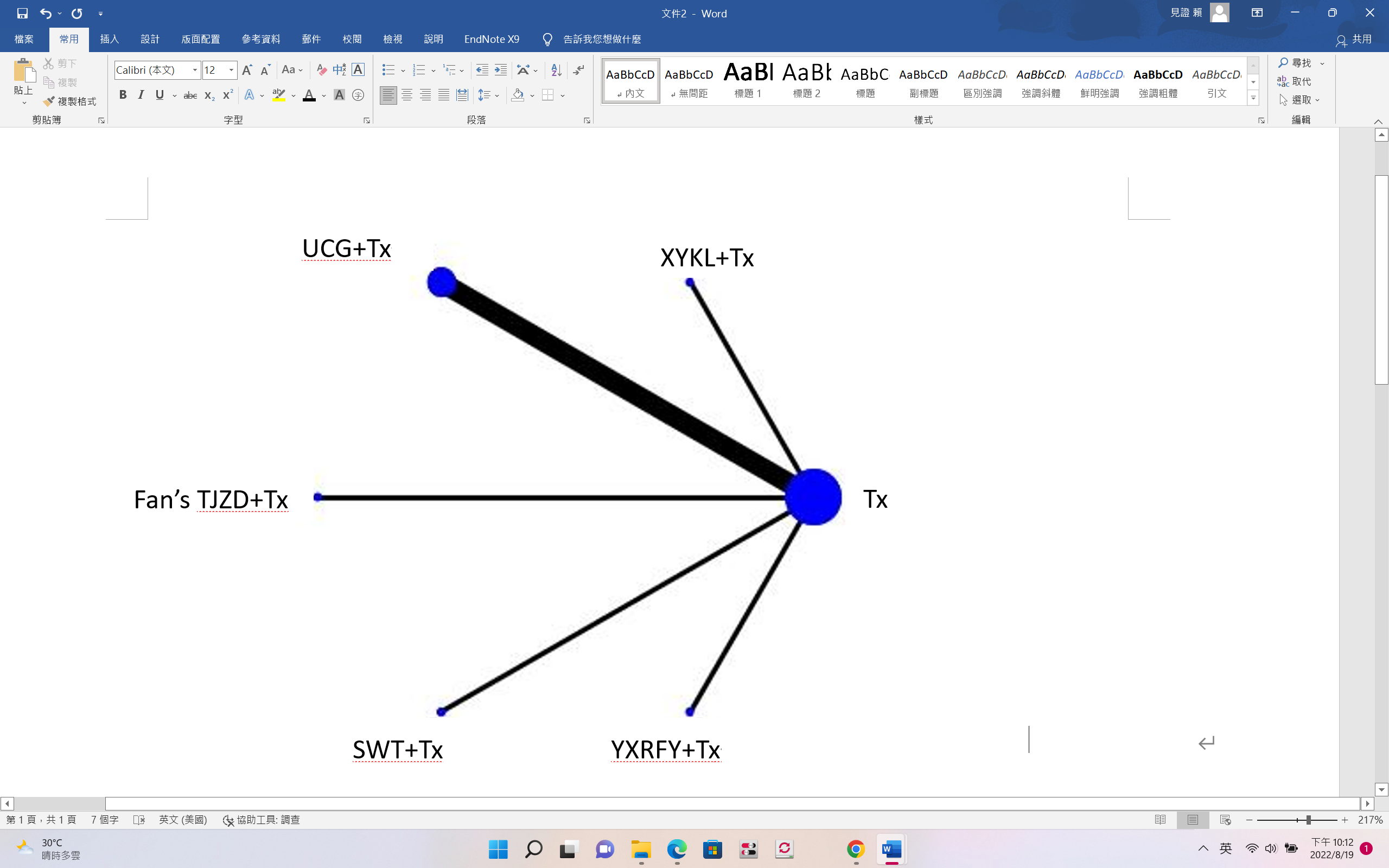


SUCRA


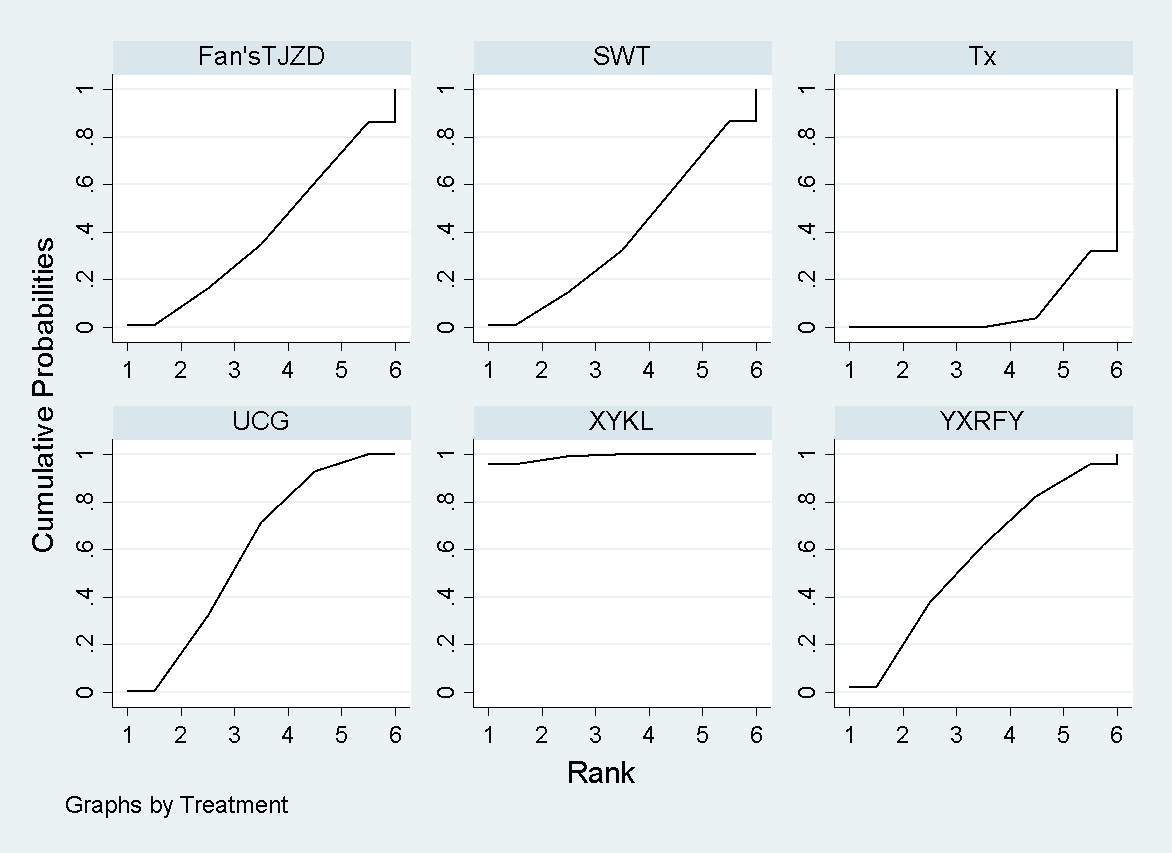


**Supplementary Figure 4. Results of network meta-analysis for** **C-reactive protein (CRP).** (a) Network plot: Each node corresponds to one treatment, node size is proportional to the number of subjects, and line thickness is proportional to the number of randomized controlled trials providing comparison data. (b) SUCRA: Surface under the cumulative ranking curve. SUCRA determines the overall ranking of each treatment. A larger area under the curve corresponds to a higher ranking. Higher ranking indicates a better treatment in the network meta-analysis. Abbreviations: *FanTJZD,* Fan’s Touxie Jiedu Zhiyang Decoction; *SWT*, Siwu Tang; *Tx*, Conventional treatment; *UCG*, Uremic Clearance Granules; *XYKL*, Xiaoyang Ke Li; *YXRFY*, Yangxue Runfu Yin


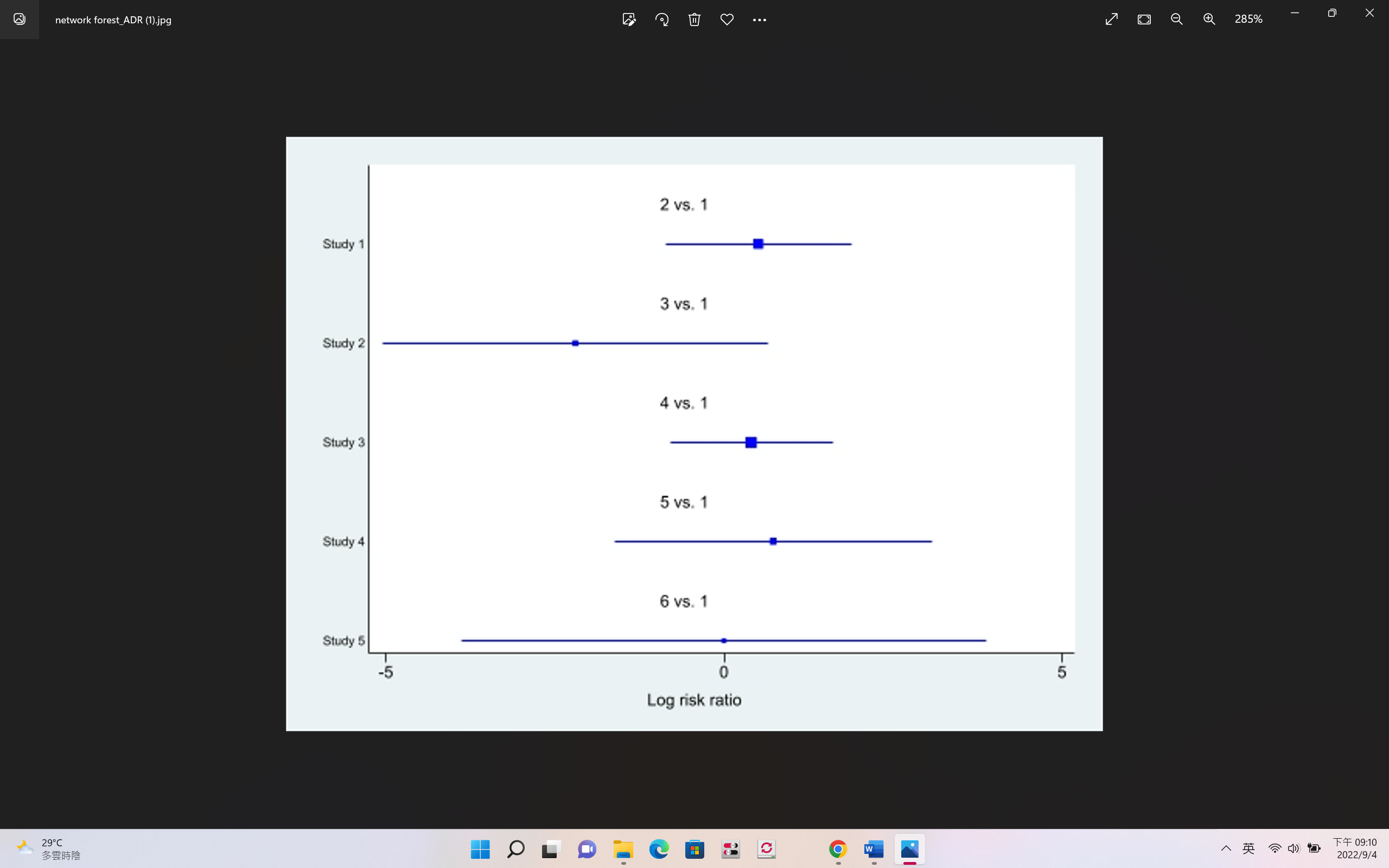


**Supplementary Figure 5. Network forest plot of adverse drug reaction (ADR)**. 1: Tx, Conventional treatment. 2: JFZYP + Tx, Jingfu Zhiyang Particles. 3: TJZD + Tx, Touxie Jiedu Zhiyang Decoction. 4: UCG + Tx, Uremic Clearance Granules. 5: BFWLT + Tx, Baifuzhi Weiliang Tang. 6: SWT + Tx, Siwu Tang.
